# Porcine Epidemic Diarrhea Virus nsp7 Inhibits MDA5 Dephosphorylation to Antagonize Type I Interferon Production

**DOI:** 10.1128/spectrum.05017-22

**Published:** 2023-03-28

**Authors:** Jiansong Zhang, Puxian Fang, Jie Ren, Sijin Xia, Huichang Zhang, Xuerui Zhu, Tong Ding, Shaobo Xiao, Liurong Fang

**Affiliations:** a State Key Laboratory of Agricultural Microbiology, College of Veterinary Medicine, Huazhong Agricultural University, Wuhan, China; b Key Laboratory of Preventive Veterinary Medicine in Hubei Province, the Cooperative Innovation Center for Sustainable Pig Production, Wuhan, China; University of Sussex

**Keywords:** porcine epidemic diarrhea virus, nonstructural protein 7, nsp7, interferon, melanoma differentiation-associated gene 5, MDA5, protein phosphatase 1, PP1

## Abstract

Porcine epidemic diarrhea virus (PEDV) is a reemerging enteropathogenic coronavirus that causes high mortality in piglets and has catastrophic effects on the global pig industry. PEDV-encoded nonstructural protein 7 (nsp7) is an important component of the viral replication and transcription complex, and a previous study reported that it inhibits poly(I:C)-induced type I interferon (IFN) production, but the mechanism by which this occurs remains unclear. Here, we demonstrated that ectopic expression of PEDV nsp7 antagonized Sendai virus (SeV)-induced interferon beta (IFN-β) production, as well as the activation of transcription factors interferon regulatory factor 3 (IRF3) and nuclear factor-kappa B (NF-κB) in both HEK-293T and LLC-PK1 cells. Mechanistically, PEDV nsp7 targets melanoma differentiation-associated gene 5 (MDA5) and interacts with its caspase activation and recruitment domains (CARDs), which sequester the interactions between MDA5 and the protein phosphatase 1 (PP1) catalytic subunits (PP1α and PP1γ), thereby suppressing MDA5 S828 dephosphorylation and keeping MDA5 inactive. Furthermore, PEDV infection attenuated MDA5 multimerization and MDA5-PP1α/-γ interactions. We also tested the nsp7 orthologs of five other mammalian coronaviruses and found that all of them except severe acute respiratory syndrome coronavirus 2 (SARS-CoV-2) nsp7 inhibited MDA5 multimerization and SeV- or MDA5-induced IFN-β production. Collectively, these results suggest that the inhibition of MDA5 dephosphorylation and multimerization may be a common strategy employed by PEDV and some other coronaviruses to antagonize MDA5-mediated IFN production.

**IMPORTANCE** Since late 2010, a reemerging porcine epidemic diarrhea virus variant with high pathogenesis has swept through most pig farms in many countries, resulting in significant economic losses. Coronavirus nonstructural protein 7 (nsp7), conserved within the family *Coronaviridae*, combines with nsp8 and nsp12 to form the viral replication and transcription complex that is indispensable for viral replication. However, the function of nsp7 in the infection and pathogenesis of coronaviruses remains largely unknown. Our present study demonstrates that PEDV nsp7 specifically competes with PP1 for binding MDA5 and impedes the PP1-mediated dephosphorylation of MDA5 at S828, thereby blocking MDA5-mediated IFN production, revealing the complex mechanism utilized by PEDV nsp7 to efficiently escape host innate immunity.

## INTRODUCTION

Porcine epidemic diarrhea virus (PEDV), an alpha coronavirus (α-CoV) that was first identified in England in the 1970s (1), generally causes an acute and highly contagious enteric viral disease in swine, characterized by vomiting, watery diarrhea, dehydration, and even death, especially in neonatal piglets (2, 3). In late 2010, a highly pathogenic PEDV variant with high morbidity and mortality rates emerged and immediately swept through many countries (4–6). At present, this PEDV variant is a leading pathogenic cause of piglet diarrhea, resulting in significant economic losses in the global pig industry.

The PEDV genome, which is approximately 28 kb long, encodes two large replicase proteins (polyprotein 1a [pp1a] and pp1b), one accessory protein (open reading frame 3 [ORF3]), and four structural proteins (spike [S], envelope [E], membrane [M], and nucleocapsid [N]) (7–9). The two large replicase proteins are cleaved by the viral papain-like protease (nonstructural protein 3 [nsp3]) and 3C-like protease (nsp5) into 16 mature nonstructural proteins (nsp1 to nsp16). Among them, nsp7, an 83-amino-acid polypeptide, is conserved within the family *Coronaviridae*. It combines with nsp8 and nsp12 to form a viral replication and transcription complex that is indispensable for viral replication (10). However, apart from being regarded as the “mortar” that stabilizes the nsp7/nsp8 complex structure, the roles of nsp7 in the infection and pathogenesis of coronaviruses remain largely unknown.

Innate immunity is the first line of host defense against viral infections. The RIG-I-like receptors (RLRs) retinoic acid-inducible gene I (RIG-I) and melanoma differentiation-associated gene 5 (MDA5) are crucial cytoplasmic pattern recognition receptors (PRRs) for the recognition of viral RNA. Under normal physiological conditions, RIG-I and MDA5 are kept in autorepressed states by constitutive phosphorylation. Upon virus infection or viral RNA binding, the phosphorylated states of RIG-I and MDA5 are quickly released by the protein phosphatase 1 (PP1) catalytic subunits (PP1α and PP1γ), thus triggering the activation of these proteins. Subsequently, activated RIG-I and MDA5 interact with the mitochondrial antiviral signaling protein (MAVS, also called VISA, IPS-1, or Cardif) to form a complex, consequently resulting in the activation of transcription factors nuclear factor-kappa B (NF-κB), AP1, and interferon regulation factor 3 and 7 (IRF3/7), followed by the production of type I interferon (IFN-I) (11–14). To establish efficient infection, many viruses, including coronaviruses (CoVs), encode numerous immunomodulatory proteins that inhibit IFN-I production, such as severe acute respiratory syndrome coronavirus 2 (SARS-CoV-2) ORF6, ORF7, ORF8, ORF9b, M, S, and N proteins (15, 16), Middle East respiratory syndrome coronavirus (MERS-CoV) ORF4a, ORF4b, and ORF5 proteins (17–19), and porcine delta coronavirus (PDCoV) NS6, NS7a, and N proteins (20–22). As a reemerging enteropathogenic CoV, PEDV has been confirmed to inhibit IFN-I production, and several of its proteins have been reported to be IFN-I antagonists. For example, PEDV N protein inhibits IFN-β production via sequestering the interaction between TANK-binding kinase 1 (TBK1) and IRF3 (23), PEDV E protein acts as an IFN-β antagonist through the inhibition of both RIG-I signaling-associated molecule expression and IRF3 nuclear translocation (24), PEDV M protein interacts with IRF7 to inhibit its phosphorylation and dimerization, leading to the decreased expression of IFN-I (25), PEDV nsp3 suppresses IFN-I production by removing the ubiquitinated conjugates from RIG-I and STING via its deubiquitinating activity (26), and PEDV nsp5 cleaves NEMO via its cysteine protease activity to antagonize IFN-I production (27). As for PEDV nsp7, Zhang et al. previously reported that this protein is an IFN antagonist according to the results of their screen of all PEDV-encoded proteins; however, they did not further investigate the mechanisms of how nsp7 antagonizes IFN-β production (28).

In this study, we demonstrate that PEDV nsp7 antagonizes IFN-β production via interacting with MDA5, thereby blocking the interaction of MDA5 with PP1α/γ and consequently preventing the dephosphorylation and activation of MDA5. We also found that the nsp7 orthologs of some other CoVs, particularly the swine enteric CoVs, inhibited MDA5-mediated IFN-β production.

## RESULTS

### PEDV nsp7 blocks SeV-induced IFN-β production.

**FIG 1:**
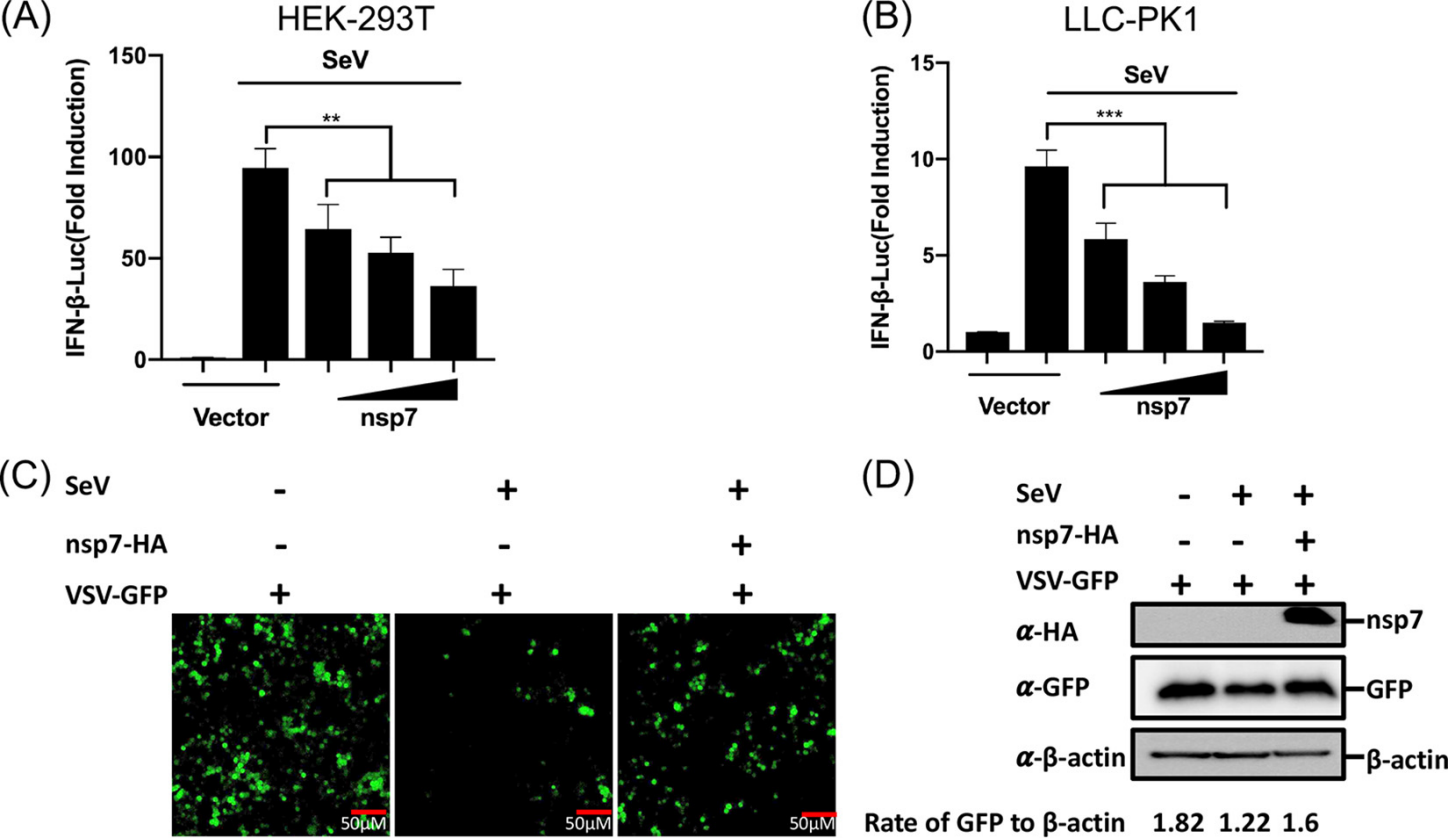
PEDV nsp7 antagonizes SeV-induced IFN-β production. (A, B) HEK-293T cells (A) or LLC-PK1 cells (B) were cotransfected with IFN-β-Luc (0.1 μg/well) and pRL-TK (0.02 μg/well) plasmids, along with an increasing amount of pCAGGS-nsp7-HA (0.2, 0.4, or 0.8 μg/well) or empty vector, followed by treatment with or without SeV (10 hemagglutination activity units/well) for 12 h. The cells were lysed and subjected to dual-luciferase assays. The firefly luciferase activity is relative to that of an untreated empty vector control, with normalization to the Renilla reniformis luciferase activity. (C, D) HEK-293T cells were transfected with pCAGGS-nsp7-HA (0.8 μg/well) or empty vector. After 24 h, the cells were infected with SeV for 12 h, and then cell supernatants were collected and subjected to UV irradiation treatment before being added to a monolayer of HEK-293T cells in a 24-well plate for 24 h. These cells were inoculated with VSV-GFP for 12 h, after which the level of viral replication was observed via fluorescence microscopy (C) and Western blot analysis (D). Target protein expression was quantitatively estimated using ImageJ software and is presented as the density value relative to that of β-actin. The results presented are representative of the mean values and standard deviations of data from three independent experiments. **, *P < *0.01; ***, *P < *0.001.

To investigate whether PEDV nsp7 inhibited IFN-β production, HEK-293T cells or LLC-PK1 cells were cotransfected with the reporter plasmids IFN-β-Luc and pRL-TK, along with increasing amounts of pCAGGS-nsp7-HA or empty vector. At 24 h after cotransfection, the cells were left untreated or infected with Sendai virus (SeV) for 12 h and then lysed to determine the IFN-β promoter-driven luciferase activity. The results showed that SeV notably induced the IFN-β promoter activation in both HEK-293T and LLC-PK1 cells; however, these inductions were significantly inhibited by nsp7 expression in a dose-dependent manner (Fig. 1A and B). To further confirm the results from our dual-luciferase reporter assay, an IFN bioassay using an IFN-sensitive vesicular stomatitis virus expressing green fluorescent protein (VSV-GFP) was performed in HEK-293T cells. As shown by the results in Fig. 1C, the cellular supernatants from SeV-infected cells remarkably hindered VSV-GFP replication; however, supernatants from cells with nsp7 expression had partially restored levels of VSV-GFP replication compared with those of cells treated with supernatants from empty vector-transfected cells. Consistent with the results shown in Fig. 1C, our Western blotting results showed that the enhanced green fluorescent protein (EGFP) expression was significantly suppressed in the cellular supernatant from SeV-infected cells but was dramatically restored in the supernatant from cells overexpressing nsp7 (Fig. 1D). These results indicate that PEDV nsp7 is an IFN antagonist.

### PEDV nsp7 impairs the activation of IRF3 and NF-κB.

**FIG 2:**
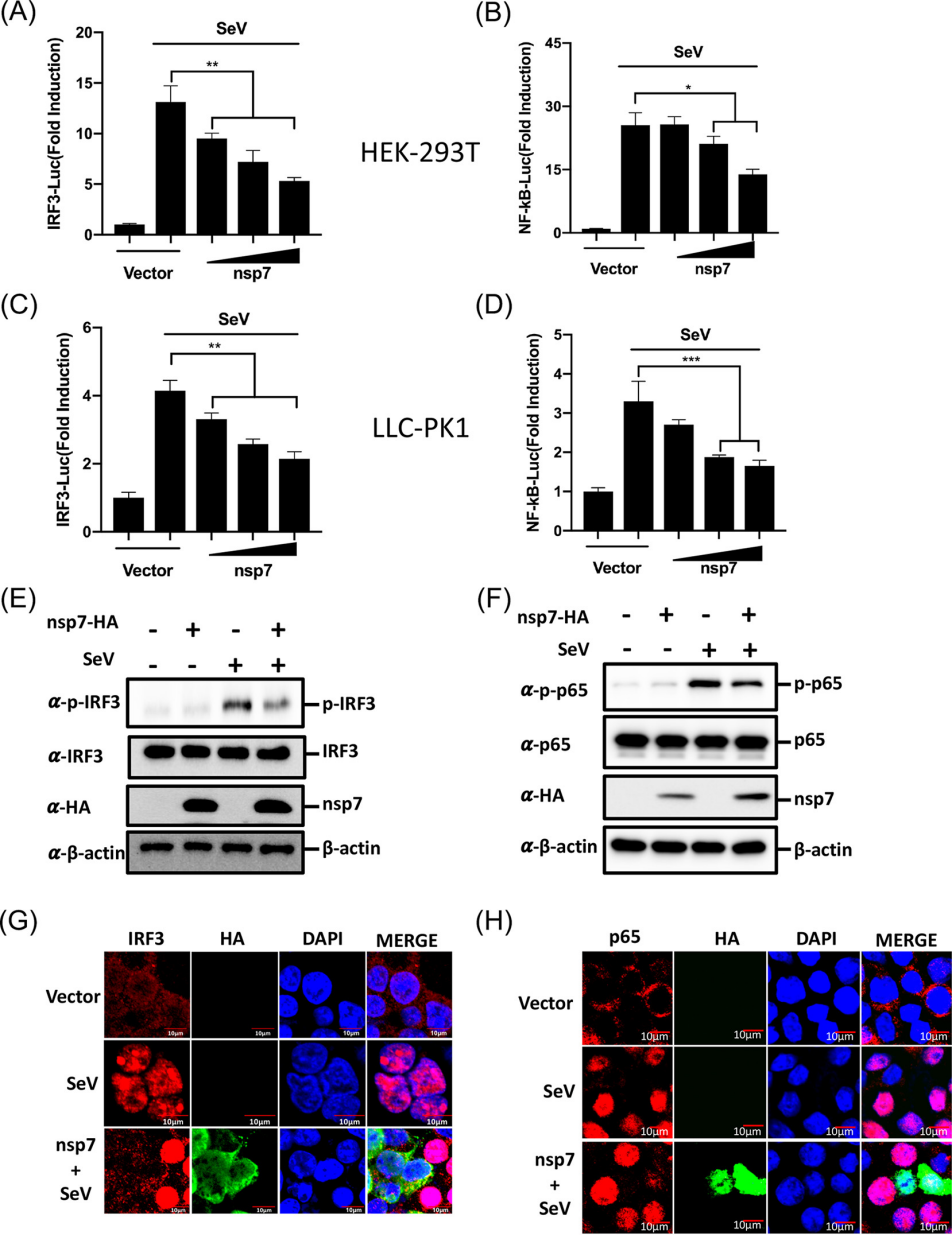
PEDV nsp7 inhibits activation of IRF3 and NF-κB. (A to D) HEK-293T cells (A and B) or LLC-PK1 cells (C and D) were cotransfected with IRF3-Luc (A and C) or NF-κB-Luc (B and D) (0.1 μg/well) and pRL-TK (0.02 μg/well) plasmids along with increasing amounts of pCAGGS-nsp7-HA (0.2, 0.4, or 0.8 μg/well) or empty vector and then treated with or without SeV for 12 h and subsequently assessed with a dual-luciferase assay. (E, F) HEK-293T cells were transfected with pCAGGS-nsp7-HA (0.8 μg/well) or empty vector for 24 h and then infected with SeV or left untreated. After 8 h, the cells were lysed and subjected to Western blot analysis with primary antibodies against phosphorylated IRF3 (p-IRF3 Ser386) and total IRF3 (E) or phosphorylated p65 (p-p65) and total p65 (F), HA, and β-actin. (G, H) HEK-293T cells were transfected with pCAGGS-nsp7-HA (0.8 μg/well) or empty vector for 24 h and then left uninfected or infected with SeV for 8 h as described for panels E and F. The cells were subsequently fixed and subjected to immunofluorescence analysis to detect nsp7 (green) and IRF3 (red) (G) or p65 (red) (H). Cell nuclei were stained with DAPI (blue). Fluorescent images were acquired with a confocal laser scanning microscope (FluoView 3.1; Olympus, Japan). The results are representative of three independent experiments performed in triplicate. Error bars show standard deviations. *, *P < *0.05; **, *P < *0.01; ***, *P < *0.001.

The binding of activated transcription factors IRF3 and NF-κB to the IFN-β promoter is a key step for the induction of IFN-β transcription (29, 30). We next explored the effect of nsp7 on the activation of IRF3 and NF-κB. To this end, HEK-293T or LLC-PK1 cells were cotransfected with the luciferase reporter plasmids IRF3-Luc or NF-κB-Luc and pRL-TK, along with pCAGGS-nsp7-HA or empty vector, and 24 h later, the cells were infected with SeV for 12 h. As shown by the results in Fig. 2, SeV infection remarkably induced the promoter activities of IRF3 and NF-κB in HEK-293T (Fig. 2A and B) and LLC-PK1 (Fig. 2C and D) cells; however, the SeV-induced promoter activities of IRF3 and NF-κB were dose dependently inhibited by nsp7.

Phosphorylation and nuclear translocation are important hallmarks of IRF3 and NF-κB activation (31–33). Thus, we further investigated the effect of nsp7 on the phosphorylation and nuclear translocation of IRF3 and the NF-κB p65 subunit in HEK-293T cells by using Western blotting and indirect immunofluorescence assays (IFAs). As expected, SeV infection notably promoted the phosphorylation of IRF3 and p65; however, the increase in phosphorylation of these proteins was remarkably impeded by nsp7 expression (Fig. 2E and F). Consistent with the Western blotting results, the nuclear translocations of IRF3 and p65 were also blocked by nsp7 expression (Fig. 2G and H). Collectively, these results further support the notion that PEDV nsp7 inhibits IFN production by blocking the activation of IRF3 and p65.

### PEDV nsp7 inhibits MDA5-mediated IFN-β production.

**FIG 3:**
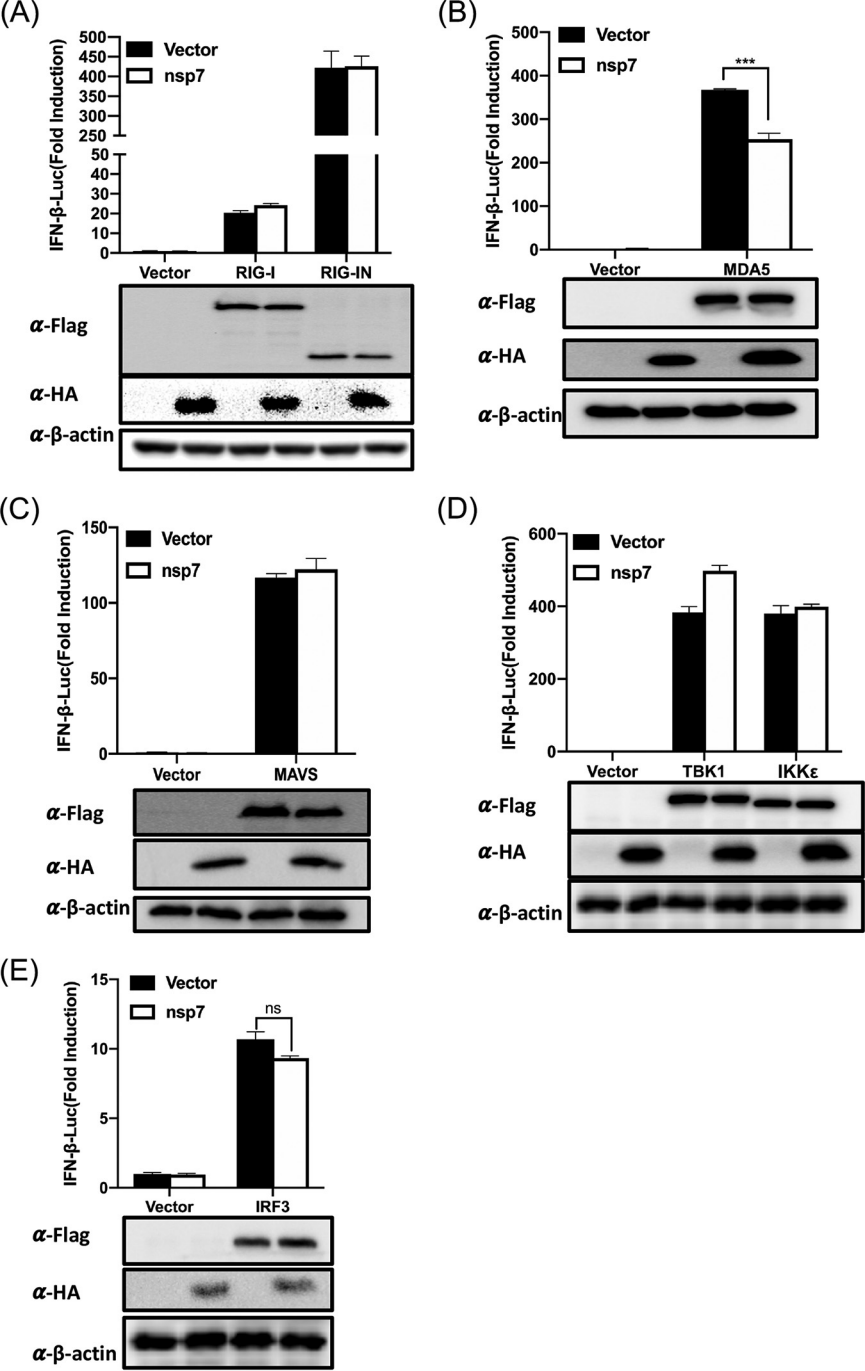
PEDV nsp7 inhibits MDA5-mediated IFN-β production. (A to E) HEK-293T cells were cotransfected with IFN-β-Luc, pRL-TK, and 0.3 μg/well of plasmid expressing Flag-tagged RIG-I, RIG-IN (A), MDA5 (B), MAVS (C), TBK1, IKKε (D), or IRF3 (E), with or without pCAGGS-nsp7-HA (0.7 μg/well) for 24 h. The cells were then lysed and subjected to dual-luciferase assays. The firefly luciferase activity is relative to that of the empty vector control, with normalization to the *Renilla reniformis* luciferase activity. The results are representative of three independent experiments performed in triplicate. Error bars show standard deviations. ***, *P < *0.001; ns, not significant.

The observation that PEDV nsp7 counteracts SeV-induced IFN-β production, as well as IRF3 and NF-κB activation, raises the possibility that nsp7 targets one or several molecules of the RLR pathway to impede IFN-I induction. To clarify this, we performed a series of dual-luciferase reporter assays by cotransfecting a plasmid expressing PEDV nsp7, reporter plasmids IFN-β-Luc and pRL-TK, and Flag-tagged MDA5, RIG-I, RIG-IN (a constitutively active form of RIG-I), MAVS, TBK1, IκB kinase subunit epsilon (IKKε), or IRF3 expression plasmids or empty vector. The results showed that all tested components of the RLR signaling pathway significantly induced activation of the IFN-β promoter. However, ectopic expression of nsp7 inhibited only the MDA5-mediated IFN-β promoter activation. In contrast, nsp7 did not obviously affect RIG-I-, RIG-IN-, MAVS-, TBK1-, IKKε-, or IRF3-mediated IFN-β promoter activation (Fig. 3A to E). These results suggest that PEDV nsp7 disrupts IFN-I production via acting on the level of MDA5.

### PEDV nsp7 interacts with MDA5 and interferes with its assembly.

**FIG 4:**
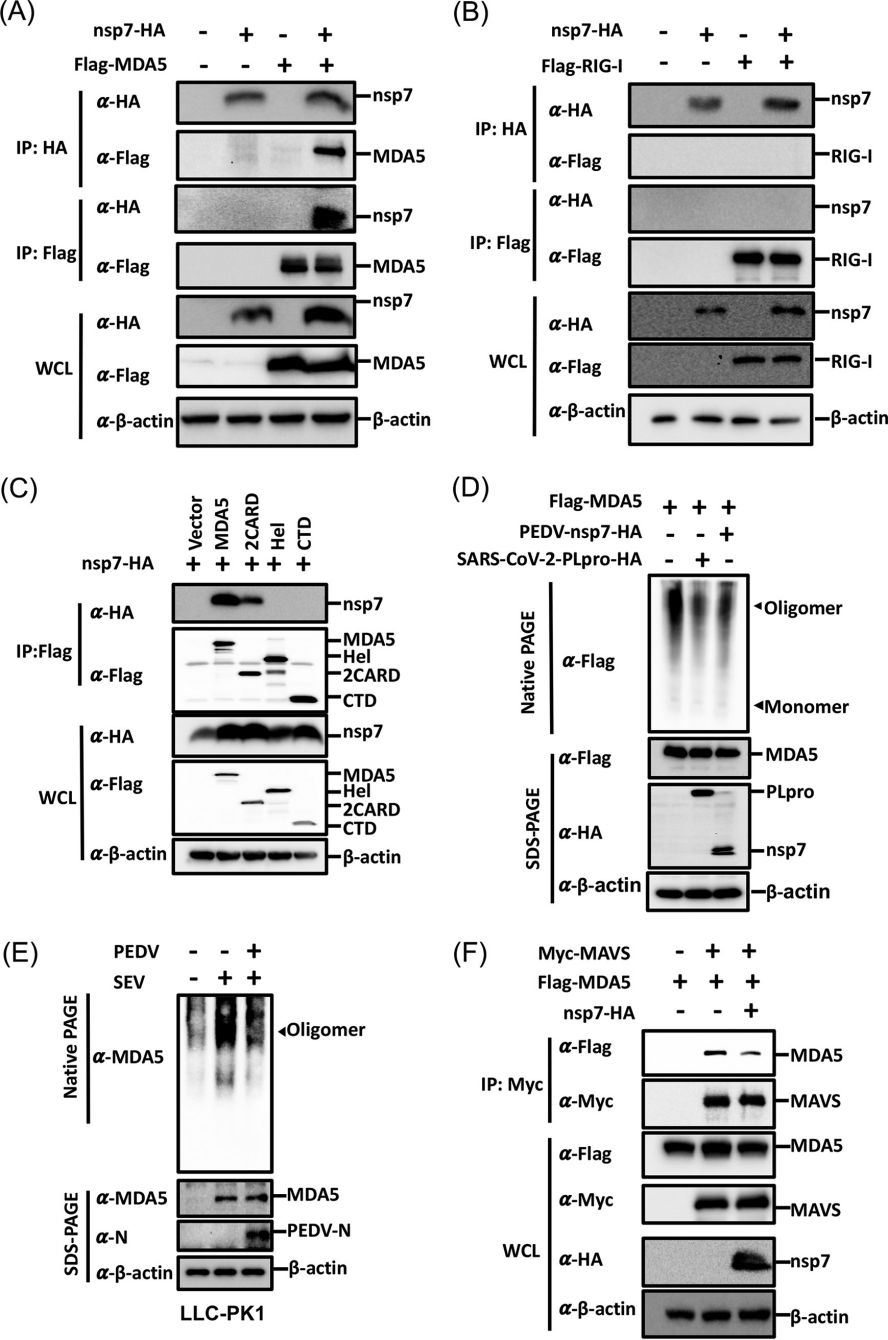
PEDV nsp7 interacts with MDA5 and interferes with its assembly. (A to C) HEK-293T cells cultured in six-well plates were transfected with pCAGGS-Flag-MDA5 (0.9 μg/well) (A) or plasmids expressing RIG-I (B) or truncated mutants of MDA5 (C) along with pCAGGS-nsp7-HA (2.1 μg/well) or empty vector. Lysates of these cells were assessed by coimmunoprecipitation (co-IP) assays with anti-HA or anti-Flag antibody. The whole-cell lysates (WCL) and immunoprecipitation (IP) complexes were analyzed by Western blotting using antibodies against Flag, HA, and β-actin. (D) HEK-293T cells cultured in a 24-well plate were cotransfected with pCAGGS-Flag-MDA5 (0.2 μg/well) and pCAGGS-nsp7-HA (1.2 μg/well), pCAGGS-SARS-CoV-2-PLpro-HA (0.8 μg/well), or empty vector. Lysates of these cells were collected, mixed with native loading buffer or SDS-PAGE loading buffer, and then respectively subjected to native PAGE or SDS-PAGE assays, followed by Western blotting using antibodies against Flag, HA, and β-actin. (E) LLC-PK1 cells cultured in a 12-well plate were pretreated with or without SeV for 6 h and then mock infected or infected with PEDV (0.1 multiplicity of infection [MOI]). After 6 h, the cells were lysed and subjected to native PAGE or SDS-PAGE, followed by Western blotting using antibodies against MDA5, PEDV-N, and β-actin. (F) HEK-293T cells cultured in six-well plates were cotransfected with pCAGGS-Myc-MAVS (0.5 μg/well) and pCAGGS-Flag-MDA5 (0.5 μg/well), along with 1.5 μg/well of pCAGGS-nsp7-HA or empty vector. Lysates of these cells were used to perform co-IP assays with anti-Myc antibody and Western blotting using antibodies against Flag, HA, Myc, and β-actin. The results are representative of three independent experiments performed in triplicate.

To determine whether MDA5 is a direct target of nsp7, a coimmunoprecipitation (co-IP) experiment examining the interaction of nsp7 with MDA5 was performed. HEK-293T cells were cotransfected with pCAGGS-nsp7-HA and pCAGGS-Flag-MDA5, and 24 h later, lysates of these cells were collected and subjected to co-IP assays with anti-hemagglutinin (HA) and anti-Flag monoclonal antibodies. The results showed that nsp7-HA interacted with Flag-MDA5 (Fig. 4A). Simultaneously, we also investigated the interaction of nsp7 with RIG-I. As expected, no interaction between nsp7 and RIG-I was observed (Fig. 4B). MDA5 is composed of two N-terminal caspase activation and recruitment domains (2CARD), an RNA helicase domain (Hel), and a C-terminal domain (CTD) (34). We further detected the key domain(s) of MDA5 that interact with nsp7 and found that nsp7 specifically interacted with the 2CARD of MDA5, rather than its Hel or CTD (Fig. 4C).

The core filament formation of MDA5 is essential for its activation and IFN induction (35, 36). Thus, we investigated the effect of nsp7 on MDA5 multimerization by performing native PAGE. The SARS-CoV-2 papain-like protease (PLpro), a known antagonist of MDA5 multimerization (37), served as a positive control in this experiment. As shown by the results in Fig. 4D, obvious MDA5 multimerization was detected in the cells transfected with pCAGGS-Flag-MDA5; however, the level of MDA5 multimerization was significantly attenuated in cells overexpressing SARS-CoV-2 PLpro or PEDV nsp7. We then assessed the endogenous MDA5 multimerization in the context of PEDV infection. As shown by the results in Fig. 4E, SeV infection significantly promoted MDA5 multimerization, but SeV-induced MDA5 multimerization was obviously inhibited after PEDV infection. MAVS is a central hub of the RLR pathway, connecting virus recognition and downstream signaling cascades, and its activation requires MDA5 multimerization (38). Therefore, we examined the effect of nsp7 on the interaction between MDA5 and MAVS. As shown by the results in Fig. 4F, an obvious interaction between MDA5 and MAVS was observed; however, this interaction was significantly weakened by nsp7 expression. Taken together, these results demonstrate that PEDV nsp7 specifically interacts with MDA5 2CARD and interferes with MDA5 multimerization, leading to inhibition of the MDA5-MAVS interaction and subsequent IFN production.

### PEDV nsp7 suppresses MDA5 S828 dephosphorylation.

**FIG 5:**
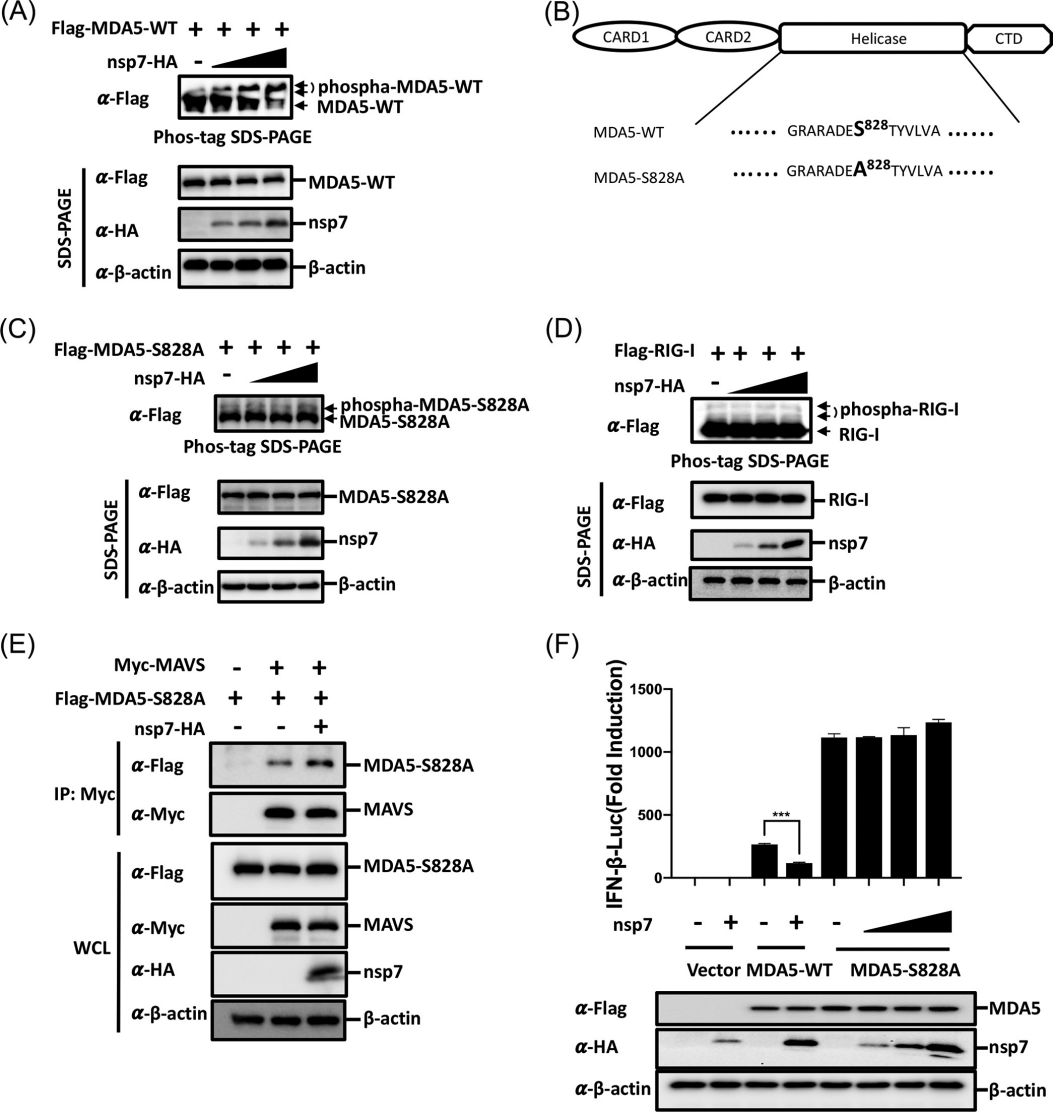
PEDV nsp7 suppresses MDA5 S828 dephosphorylation. (A) HEK-293T cells cultured in 24-well plates were cotransfected with pCAGGS-Flag-MDA5-WT (0.2 μg/well) and pCAGGS-nsp7-HA (0.8 μg/well) or empty vector for 24 h. The cells were then lysed, and their proteins were separated by Phos-tag PAGE and subjected to Western blot analysis. (B) Schematic representation of the MDA5 wild-type (WT) and S828A mutant. (C) HEK-293T cells cultured in 24-well plates were cotransfected with pCAGGS-Flag-MDA5-S828A (0.2 μg/well), along with pCAGGS-nsp7-HA (0.8 μg/well) or empty vector for 24 h. The cells were then lysed, and their proteins were separated by Phos-tag PAGE and subjected to Western blot analysis. (D) HEK-293T cells cultured in 24-well plates were cotransfected with pCAGGS-Flag-RIG-I (0.2 μg/well) and pCAGGS-nsp7-HA (0.8 μg/well) or empty vector for 24 h and then analyzed as described for panels A and C. (E) HEK-293T cells cultured in six-well plates were cotransfected with pCAGGS-Flag-MDA5-S828A (0.5 μg/well) and pCAGGS-Myc-MAVS (0.5 μg/well), along with pCAGGS-nsp7-HA (1.5 μg/well) or empty vector. Co-IP assay and Western blot analysis were performed as described in for panel F. (F) HEK-293T cells were cotransfected with reporter plasmids IFN-β-Luc and pRL-TK, with 0.2 μg/well of pCAGGS-Flag-MDA5-WT or pCAGGS-Flag-MDA5-S828A, and with increasing amounts of pCAGGS-nsp7-HA (0.2, 0.4, or 0.8 μg/well) or empty vector. After 24 h, the cells were lysed and subjected to dual-luciferase assays. Results are representative of three independent experiments performed in triplicate. Error bars show standard deviations. ***, *P < *0.001.

Previous studies revealed that MDA5 S828 is phosphorylated by RIO kinase 3 (RIOK3, a protein kinase), which inhibits MDA5 multimerization and keeps this protein in a quiescent condition; however, once virus infection or viral RNA binding occurs, MDA5 is quickly activated via dephosphorylation at S828 by PP1 (39). We hypothesized that PEDV nsp7 modulates MDA5 S828 dephosphorylation to prevent MDA5 multimerization. To investigate this possibility, we constructed the phospho-null MDA5-S828A expression plasmid (bearing a mutation of S to A at position 828 of MDA5) and performed phosphate affinity SDS-PAGE to detect wild-type MDA5 (MDA5-WT) and MDA5-S828A phosphorylation in the presence and absence of nsp7 expression. As shown by the results in Fig. 5A, overexpressing nsp7 notably enhanced the expression of phosphorylated MDA5-WT in a dose-dependent manner, suggesting that nsp7 inhibited MDA5 dephosphorylation. In contrast, the phosphorylation of MDA5-S828A was barely affected by nsp7 (Fig. 5B and C). We also tested the effect of nsp7 on RIG-I phosphorylation and found that PEDV nsp7 had no obvious effect on the level of RIG-I phosphorylation (Fig. 5D), reconfirming that PEDV nsp7 specifically inhibited the dephosphorylation of MDA5. Furthermore, we examined the effect of nsp7 on the binding of MAVS to MDA5-S828A by performing a competition assay. As shown by the results in Fig. 5E, nsp7 had no significant inhibitory effect on the MAVS–MDA5-S828A interaction. To further confirm the above-described results, a dual-luciferase assay was performed to detect the effect of nsp7 on MDA5-S828A-mediated IFN-β promoter activation. As expected, MDA5-S828A induced a higher level of IFN-β promoter activation than did MDA5-WT, which is consistent with the results of previous work (39). nsp7 expression notably suppressed the IFN-β promoter activation induced by MDA5-WT but had no obvious inhibitory effect on MDA5-S828A-induced IFN-β promoter activation (Fig. 5F). Together, these results indicate that the inhibition of the MDA5–MAVS interaction by nsp7 is a direct effect of its ability to block MDA5 S828 dephosphorylation.

### PEDV nsp7 competes with PP1α/γ for binding to MDA5.

**FIG 6:**
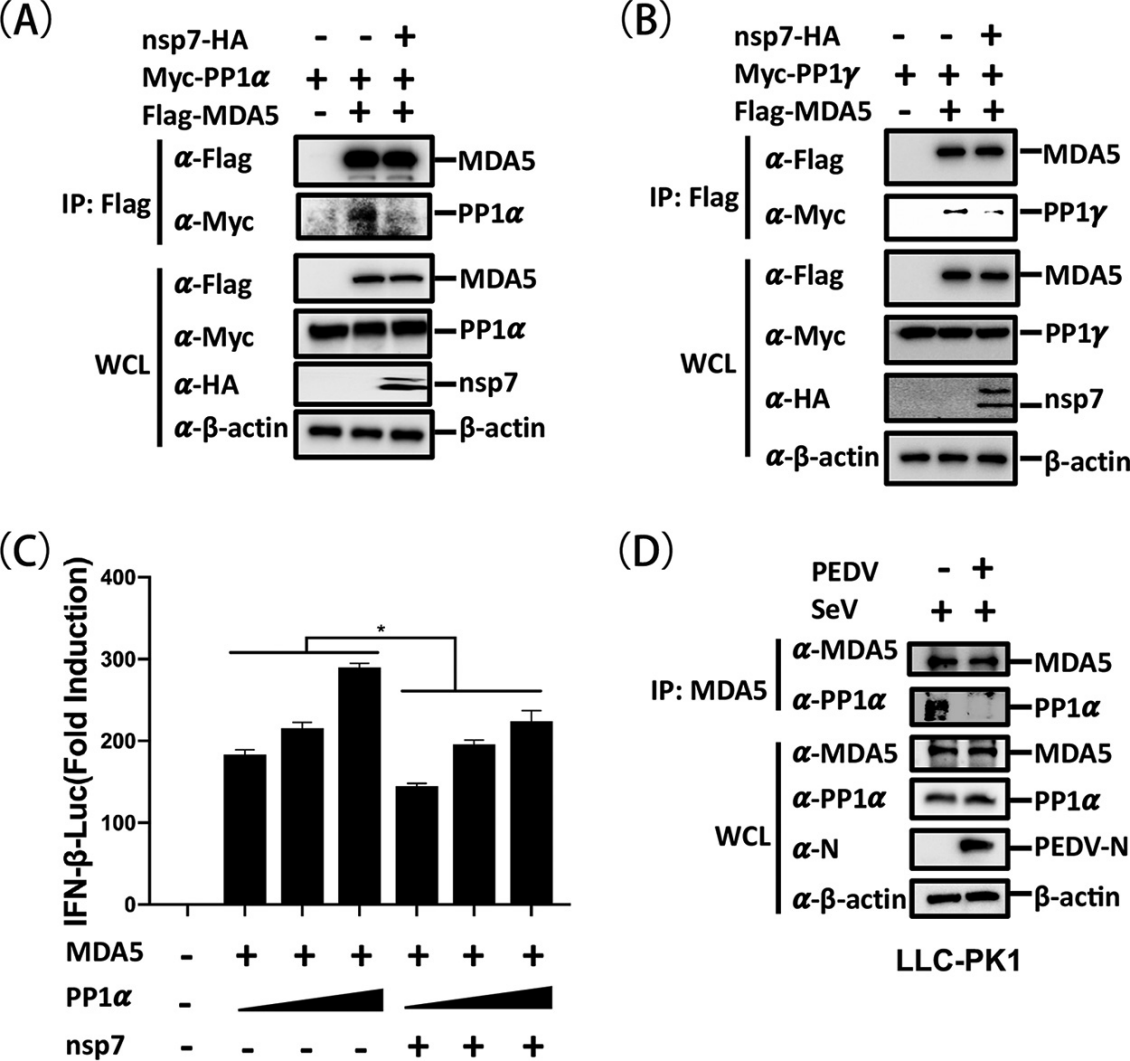
PEDV nsp7 competes with PP1α/γ for binding to MDA5. (A, B) HEK-293T cells cultured in six-well plates were cotransfected with 0.5 μg/well of pCAGGS-Myc-PP1α or pCAGGS-Myc-PP1γ and pCAGGS-Flag-MDA5 (0.5 μg/well) with or without pCAGSS-nsp7-HA (1.5 μg/well). At 24 h posttransfection, the cells were lysed and subjected to co-IP assay using anti-Flag antibody and a subsequent Western blot analysis. (C) HEK-293T cells were cotransfected with reporter plasmids IFN-β-Luc and pRL-TK, 0.2 μg/well of pCAGGS-Flag-MDA5, and increasing amounts of pCAGGS-Myc-PP1α (0.05, 0.1, or 0.2 μg/well), with or without pCAGGS-nsp7-HA (0.6 μg/well). After 24 h, the cells were lysed and assessed by dual-luciferase assays. (D) LLC-PK1 cells cultured in 60-mm dishes were stimulated with or without SeV for 6 h, followed by no treatment or PEDV infection (MOI = 0.1). At 6 h postinfection, the cells were lysed and used for a co-IP assay with anti-MDA5 antibody, followed by Western blotting using anti-MDA5, anti-PP1α, anti-PEDV-N, and anti-β-actin antibodies. The results are representative of three independent experiments performed in triplicate. Error bars show standard deviations. *, *P < *0.05.

Upon viral infection, MDA5 can recruit and interact with PP1α/γ via its exposed CARDs, promoting its own dephosphorylation and activation (14). Because nsp7 interacts with the 2CARD of MDA5 and inhibits MDA5 dephosphorylation, we speculated that nsp7 competes with PP1α/γ for binding to MDA5. To test this hypothesis, HEK-293T cells were cotransfected with pCAGGS-Flag-MDA5 and pCAGGS-Myc-PP1α or -PP1γ, along with pCAGGS-nsp7-HA or empty vector, and then assessed in competitive co-IP assays with an anti-Flag antibody. As shown in Fig. 6A and B, the results demonstrated that both PP1α and PP1γ could be coimmunoprecipitated by MDA5 but that the ectopic expression of nsp7 remarkably reduced the MDA5-PP1α or MDA5-PP1γ interactions. Using PP1α as a representative PP1, we performed a dual-luciferase assay to evaluate the effect of nsp7 on PP1α-mediated MDA5 activation. The results showed that PP1α significantly increased the level of MDA5-mediated IFN-β promoter activation in a dose-dependent manner, but this increase was inhibited by nsp7 (Fig. 6C). We also examined the endogenous MDA5-PP1α interaction under the condition of PEDV infection. The results showed that an obvious interaction between MDA5 and PP1α was detected in LLC-PK1 cells following SeV stimulation. However, this interaction was significantly inhibited after PEDV infection (Fig. 6D). These results demonstrate that PEDV nsp7 competes with PP1α/γ for binding to MDA5.

### MDA5 is a common target of nsp7 proteins of swine enteric CoVs.

**FIG 7:**
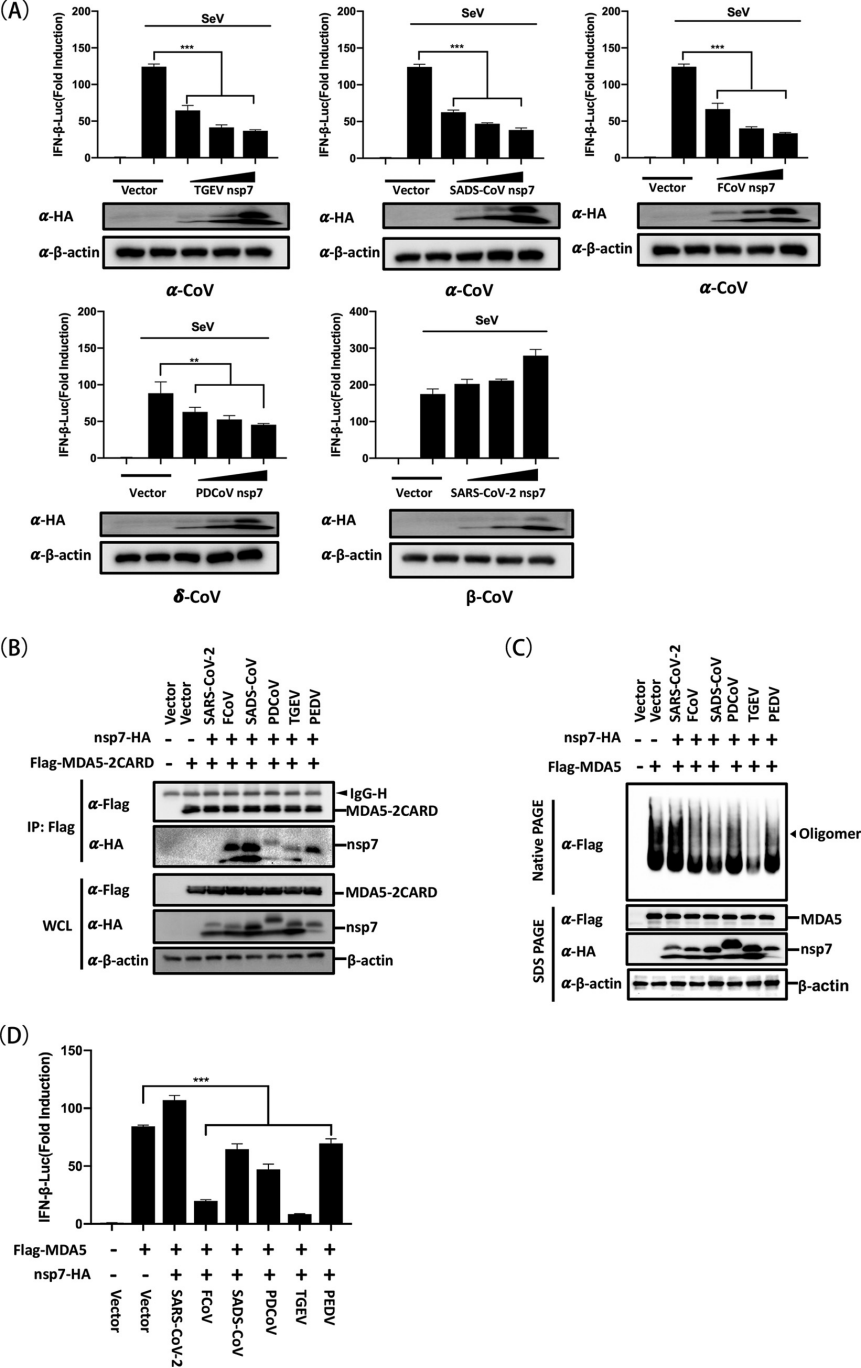
MDA5 is a common target of the nsp7 proteins of swine enteric CoVs. (A) HEK-293T cells were cotransfected with the reporter plasmids IFN-β-Luc and pRL-TK, along with increasing amounts (0.2, 0.4, or 0.8 μg/well) of an expression plasmid encoding an HA-tagged nsp7 from one of several different CoVs (TGEV, SADS-CoV, FCoV, PDCoV, or SARS-CoV-2) or empty vector. At 24 h posttransfection, the cells were treated with or without SeV (10 hemagglutination activity units/well) for 12 h and then subjected to dual-luciferase assays. (B) HEK-293T cells were cotransfected with pCAGGS-Flag-MDA5-2CARD (0.5 μg/well) and an expression plasmid encoding an HA-tagged nsp7 from one of several different CoVs (2.0 μg/well) or empty vector. At 24 h posttransfection, lysates of these cells were assessed by co-IP assay with anti-Flag antibody and a subsequent Western blot analysis. (C, D) HEK-293T cells were cotransfected with the reporter plasmids IFN-β-Luc and pRL-TK, pCAGGS-Flag-MDA5, and an expression plasmid encoding an HA-tagged nsp7 from one of several different CoVs or empty vector. After 24 h, the cells were lysed and subjected to a native PAGE assay for detecting MDA5 multimerization (C) and dual-luciferase assays (D). The results are representative of three independent experiments performed in triplicate. Error bars show standard deviations. **, *P < *0.01; ***, *P < *0.001.

To determine whether nsp7 orthologs from different mammalian CoVs target MDA5 to inhibit IFN production, we selected three other α-CoVs (transmissible gastroenteritis virus [TGEV], swine acute diarrhea syndrome coronavirus [SADS]-CoV, and feline coronavirus [FCoV]), one β-CoV (SARS-CoV-2), and one δ-CoV (PDCoV). HEK-293T cells were cotransfected with the reporter plasmids IFN-β-Luc and pRL-TK, along with increasing amounts of expression plasmids encoding an HA-tagged nsp7 from one of the selected mammalian CoVs, for 24 h and then infected with SeV for 12 h before being assessed by dual-luciferase activity assays. The results showed that ectopic expression of nsp7 from TGEV, SADS-CoV, FCoV, or PDCoV, but not nsp7 from SARS-CoV-2, considerably inhibited SeV-induced IFN-β promoter activation in a dose-dependent manner (Fig. 7A). Analogously, except for the nsp7 from SARS-CoV-2, the nsp7 from each of the mammalian CoVs tested interacted with MDA5 2CARD and significantly suppressed both MDA5 multimerization and MDA5-mediated IFN-β promoter activation (Fig. 7B to D), suggesting that the inhibition of MDA5 multimerization by nsp7 is a mechanism conserved at least across swine enteric CoVs to antagonize MDA5-mediated IFN production.

## DISCUSSION

The viral antagonism of host innate immune responses is critical for virus replication and often determines the outcome of an infection. Coronaviruses have evolved various strategies to evade the host innate immune response, including antagonizing IFN production, inhibiting IFN signaling, and enhancing IFN resistance (40–42). Our previous work demonstrated that PEDV nsp7 antagonizes the IFN-induced JAK-STAT signaling pathway by blocking ISGF3 nuclear translocation (43). In the present study, we confirmed that PEDV nsp7 interacts with MDA5 and impedes the PP1-mediated dephosphorylation of MDA5 at S828, consequently blocking MDA5-mediated IFN production, revealing the complex mechanism utilized by PEDV nsp7 to efficiently escape host innate immunity.

RIG-I and MDA5 are important cytoplasmic sensors for the recognition of viral double-stranded RNA (dsRNA) to initiate the IFN-I response. Under quiescent conditions, RIG-I and MDA5 are each maintained in an inactive state by phosphorylation at specific serine/threonine residues, such as RIG-I S8 and T170 and MDA5 S88 and S828. Upon viral RNA binding, RIG-I and MDA5 are each dephosphorylated by PP1α/γ, allowing RLR interaction with MAVS and subsequent IFN induction (44). Many viruses have developed elaborate strategies to target RIG-I and/or MDA5 as a means of antagonizing IFN production, such as West Nile virus NS1, PDCoV NS6, MERS-CoV N, influenza virus NS1, encephalomyocarditis virus 2C, and SARS-CoV-2 nsp3 (22, 37, 45–48). Here, we found that overexpressed PEDV nsp7 specifically interacted with MDA5, rather than RIG-I, and inhibited its activation (Fig. 4). Although RIG-I and MDA5 have similar domain structures, the molecular mechanisms for dsRNA recognition used by MDA5 and RIG-I are different (49). We further investigated the effect of nsp7 on the dephosphorylation of MDA5 and RIG-I and found that nsp7 specifically suppressed the level of dephosphorylation of MDA5 but not that of RIG-I (Fig. 5A and D), in agreement with previous results from dual-luciferase and co-IP assays (Fig. 3 and 4, respectively). Previous work demonstrated that the PP1-mediated dephosphorylation of MDA5 at S828 occurs prior to that of MDA5 at S88 (39). Thus, we introduced an S828A substitution in MDA5 and performed experiments to determine whether MDA5 S828 is a key dephosphorylation site. Our results showed that nsp7 did not attenuate either the interaction of MDA5-S828A with MAVS or MDA5-S828A-mediated IFN-β promoter activation, indicating that the S828 of MDA5 plays a key role in the nsp7-mediated inhibition of its dephosphorylation (Fig. 5E and F). A previous study revealed that the measles virus V protein acts as a substrate that competes with MDA5 for PP1 binding, thus blocking the dephosphorylation of MDA5 at S88 (50). Our data showed that ectopic expression of PP1α could further enhance MDA5-S828A-mediated IFN-β promoter activation (data not shown), suggesting that MDA5-S828A still retained the ability to be dephosphorylated at S88 by PP1. However, PEDV nsp7 had no negative effect on MDA5-S828A-mediated signaling activation, implying that PEDV nsp7 did not suppress MDA5 S88 dephosphorylation. The interaction of PEDV nsp7 with MDA5 2CARD makes it possible that this viral protein competes with PP1 for binding to MDA5. Indeed, the results from competition binding experiments support this hypothesis in the context of an overexpression system and virus infection (Fig. 6). Taken together, these results suggest that PEDV nsp7 specifically inhibits MDA5 dephosphorylation at S828, which identifies an important mechanism of MDA5 inhibition distinct from that reported previously.

In the present study, our results provide evidence that the nsp7 orthologs from the four tested swine enteric CoVs (PEDV, TGEV, SADS-CoV, and PDCoV) each have obvious inhibitory effects on both the IFN-β promoter activation and MDA5 multimerization induced by SeV or MDA5 (Fig. 7A). Thus, the inhibition of MDA5 multimerization by nsp7 is a mechanism conserved at least across swine enteric CoVs. Notably, FCoV nsp7 also suppressed both SeV- and MDA5-induced IFN-β promoter activation. PEDV, TGEV, SADS-CoV, and FCoV are all members of the genus α-CoV. We speculate that the inhibition of MDA5 activation by nsp7 may be a conserved mechanism across α-CoVs; however, because the number of different α-CoV nsp7 proteins tested here is limited, more nsp7 orthologs from other α-CoVs need to be assessed to verify this hypothesis. Interestingly, the nsp7 from SARS-CoV-2 did not possess this inhibitory ability (Fig. 7), a finding that is in agreement with previous studies (51, 52), and similar results were observed for the nsp7 of mouse hepatitis virus (MHV), another β-CoV (data not shown). Whether the failure of nsp7 orthologs from β-CoVs to antagonize IFN production is conserved awaits confirmation by further study. CoV nsp7 proteins share similar overall topological structures, along with a highly conserved function as an accessory subunit of the replication transcription complex in the viral replication cycle (53, 54). Thus, the detailed mechanism underlying this difference in IFN-antagonistic ability is interesting and deserves further exploration.

CoV nsp7 and nsp8 are always inseparable, forming a heterotrimer or hexadecamer as a cofactor to enhance nsp12 RNA polymerase activity (53, 55, 56). In the present work, we also compared the inhibitory effects of expressing nsp7 alone or with nsp8 on SeV-induced IFN-β promoter activation. Interestingly, the ectopic expression of nsp8 alone had no obvious effect on the level of SeV-induced IFN-β promoter activation, and the coexpression of nsp7 with nsp8 also did not enhance the level of inhibition compared with the overexpression of nsp7 alone (data not shown). In combination with the results of our previous study, our data indicate that PEDV nsp7 suppresses IFN-I production and JAK-STAT signaling independently of nsp8. Certainly, additional experiments need to be performed to verify these speculations in the context of viral infection. In conclusion, our study demonstrates that PEDV nsp7 inhibits MDA5-mediated IFN-I production by competing with PP1α/γ for binding to MDA5 and blocking MDA5 dephosphorylation. This study reveals that the inhibition of MDA5 multimerization may be a common strategy used by swine enteric CoVs to inhibit host innate immune responses, enriching the immune regulation function of coronavirus nsp7.

## MATERIALS AND METHODS

### Cells, viruses, and reagents.

Human embryonic kidney cells (HEK-293T) and porcine kidney epithelial cells (LLC-PK1) were maintained in Dulbecco’s minimum essential medium (DMEM) and minimum essential medium (MEM), respectively, supplemented with 10% fetal bovine serum at 37°C in a humidified 5% CO_2_ incubator. PEDV strain AJ1102 (GenBank accession no. JX188454.1), transmissible gastroenteritis virus (TGEV) strain WH1 (GenBank accession no. HQ462571), PDCoV strain CHN-HN-2014 (GenBank accession no. KT336560), and swine acute diarrhea syndrome coronavirus (SADS-CoV) strain CHN-GD-2017 (GenBank accession no. MH539766) were described previously (57). Feline coronavirus (FCoV) strain 79-1146_CA (GenBank accession no. MW030109.1) was kindly provided by Guiqing Peng (Huazhong Agricultural University, Wuhan, China). Vesicular stomatitis virus expressing green fluorescent protein (VSV-GFP) was generously gifted by Zhigao Bu at Harbin Veterinary Research Institute, Chinese Academy of Agricultural Sciences, Harbin, China. Sendai virus (SeV) was obtained from the Centre of Virus Resource and Information, Wuhan Institute of Virology, Chinese Academy of Sciences, Wuhan, China. Antibodies against phosphorylated (p)-IRF3, p65, and β-actin were purchased from ABclonal (Wuhan, China). Rabbit anti-p-p65 and anti-IRF3 antibodies were purchased from Cell Signaling Technology (Danvers, MA, USA). Mouse anti-Flag, anti-GFP, anti-Myc, and anti-hemagglutinin (HA) antibodies were purchased from Medical and Biological Laboratories (Nagoya, Japan). Horseradish peroxidase (HRP)-conjugated secondary antibodies and DAPI (4′,6-diamidino-2-phenylindole) were purchased from Beyotime (Shanghai, China). DyLight 488-conjugated goat anti-mouse IgG and DyLight 594-conjugated goat anti-rabbit IgG were obtained from Abbkine (Wuhan, China).

### Plasmid constructs and transfection.

The eukaryotic expression plasmid encoding PEDV nsp7 (pCAGGS-nsp7-HA) was described previously (43). The cDNAs of the nsp7 proteins from TGEV, SADS-CoV, FCoV, and PDCoV were each amplified using specific primers and cloned into pCAGGS-HA, with an HA tag at the C terminus, resulting in the expression constructs pCAGGS-TGEV-nsp7-HA, pCAGGS-SADS-CoV-nsp7-HA, pCAGGS-FCoV-nsp7-HA, and pCAGGS-PDCoV-nsp7-HA, respectively. The cDNAs encoding SARS-CoV-2 nsp7 and PLpro (amino acids 746 to 1,060) were synthesized by Tsingke Biotechnology (China) and cloned into the pCAGGS-HA vector, generating the expression constructs pCAGGS-SARS-CoV-2-nsp7-HA and pCAGGS-SARS-CoV-2-PLpro-HA, respectively. The full-length cDNA fragments of PP1α and PP1γ were amplified from HEK-293T cells and cloned into a pCAGGS vector containing a Myc tag at the N terminus, producing pCAGGS-Myc-PP1α and pCAGGS-Myc-PP1γ, respectively. The luciferase reporter plasmids IFN-β-Luc, 4×PRDIII/I-Luc (referred to as IRF3-Luc), and 4×PRDII-Luc (referred to as NF-κB-Luc), plasmid pRL-TK (as an internal control), and expression constructs encoding Flag-tagged RIG-I, MDA5, MAVS, TBK1, IKKε, and IRF3 have been described previously (23). The full-length cDNA of MAVS was amplified using a Flag-tagged MAVS expression construct as the template and subsequently cloned into pCAGGS-Myc, generating the expression construct pCAGGS-Myc-MAVS. The expression plasmids of MDA5 truncation mutants (pCAGGS-Flag-2CARD, pCAGGS-Flag-Hel, and pCAGGS-Flag-CTD) have also been described previously (22). The plasmid pCAGGS-Flag-MDA5-S828A containing an unphosphorylated mutant form of MDA5 was generated using PCR-mediated site-directed mutagenesis to introduce the S828A substitution. All primers used in this study are listed in Table 1. All expression constructs were validated by DNA sequencing. For the transfection assay, these plasmids were transfected into cells using Jetprime transfection regent (Polyplus) in accordance with the manufacturer’s protocol.

**TABLE 1:** Primers used for the construction of plasmids

Primer	Sequence (5′–3′)
FCoV-nsp7-F	CGGGAATTCGCCACCATGTCTAAACTTACAGAGATGAAGTG
FCoV-nsp7-R	CCGGTAAGATCTCTGTAAAATAGTGGTGTTCTC
TGEV-nsp7-F	CGGGAATTCGCCACCATGTCAAAACTTACAGAGATGAAATG
TGEV-nsp7-R	GTAAGATCTCTGGAGTATGGTGGTGTTCTCAAAG
SADS-CoV-nsp7-F	CGGGAATTCGCCACCATGTCTAAACTTACTGATCTTAAGTGTG
SADS-CoV-nsp7-R	GTAAGATCTTTGAAGCACATTTCTGTTTTCAAAG
PDCoV-nsp7-F	CGGGAATTCGCCACCATGAAAATTCTTGATGCAAAAG
PDCoV-nsp7-R	GTAAGATCTCTGAACAACAGCTTTGTTCTCAAG
PP1α-F	CCGGAATTCATGTCCGACAGCGAGAAGCTCAACC
PP1α-R	CCGCTCGAGCTATTTCTTGGCTTTGGCGGAATTG
PP1γ-F	CCGGAATTCATGGCGGATTTAGATAAACTCAACATC
PP1γ-R	CCGCTCGAGCTATTTCTTTGCTTGCTTTGTGATC

### Luciferase reporter assay.

HEK-293T or LLC-PK1 cells cultured in 24-well plates were cotransfected with the indicated plasmids along with 0.1 μg/well reporter plasmid (IFN-β-Luc, NF-κB-Luc, or IRF3-Luc) and 0.02 μg/well pRL-TK for normalization, and 24 h later, they were infected with SeV (10 hemagglutination activity units/well). After 12 h, the cells were lysed, and the firefly and *Renilla* luciferase activities were determined through the dual-luciferase reporter assay system in accordance with the manufacturer’s instructions (Promega, Madison, WI).

### IFN bioassay.

The IFN bioassay was performed in HEK-293T cells by using VSV-GFP as described previously (22, 58).

### Co-IP and Western blot analysis.

Cells were transfected with plasmids or infected with virus for the indicated amounts of time and then lysed with a lysis buffer (50 mM Tris-HCl [pH 7.4], 150 mM NaCl, 1% NP-40, 10% glycerin, 0.1% SDS, 2 mM Na_2_EDTA) supplemented with a protease inhibitor cocktail (Roche) for 30 min at 4°C. Lysates were collected and centrifuged at 12,000 rpm at 4°C for 10 min. A portion of each supernatant from the lysed cells was used in the whole-cell-extract assay. The remaining portion of each supernatant was subjected to coimmunoprecipitation with affinity antibody at 4°C overnight, followed by the addition of protein A+G agarose beads (Beyotime) for another 4 h. The beads were washed three times with lysis buffer and then subjected to Western blot analysis.

For Western blotting, the protein samples separated by SDS-PAGE were transferred onto polyvinylidene difluoride (PVDF) membranes (Millipore, Darmstadt, Germany). The membranes were blocked with 10% nonfat milk in phosphate-buffered saline (PBS) with 0.1% Tween 20 (PBST) for 2 h and then incubated with the indicated primary antibodies. Two hours later, the membranes were washed three times with PBST and then incubated with the appropriate HRP-conjugated secondary antibodies. After 45 min, the membranes were washed three times and then visualized via enhanced chemiluminescence reagents (Bio-Rad, Hercules, CA, USA).

### Phosphate affinity SDS-PAGE.

Phosphate-affinity SDS-PAGE was performed by using Phos-tag SDS-PAGE as described previously (59). Briefly, cell lysates were prepared using cell lysis buffer without inhibitors (Beyotime). The proteins were separated using Phos-tag SDS-PAGE that was performed with 7% polyacrylamide gels containing 50 μM Phos-tag AAL-107 (Wako) and 100 μM MnCl_2_ under constant-current conditions (25 mA/gel). The gels were equilibrated with transfer buffer supplemented with 10 mM EDTA via three 10-min washes with shaking and then equilibrated with transfer buffer lacking EDTA via an additional 10-min wash before being subjected to a standard Western blotting procedure.

### Native PAGE.

Cells were lysed with 5× lysis buffer (Promega) on ice for 30 min and then centrifuged at 12,000 rpm at 4°C for 10 min to remove the insoluble fraction. The whole-cell extracts mixed with 5× native PAGE sample loading buffer (Beyotime) were subjected to 4% to 20% native polyacrylamide gels (Beyotime) and Western blot analysis with anti-Flag or anti-MDA5 antibody (Proteintech, Wuhan, China).

### Indirect immunofluorescence assay (IFA).

HEK-293T cells seeded onto coverslips were transfected with the indicated expression plasmids. After 24 h, the cells were left uninfected or were infected with SeV; 8 h later, they were fixed with 4% paraformaldehyde and then permeated with cold methanol. Nonspecific antibody binding was blocked with 5% bovine serum albumin. The cells were then incubated with the indicated primary antibodies, followed by treatment with fluorochrome-conjugated secondary antibodies. After being stained with DAPI, the cells were covered with coverslips, and their fluorescence was visualized with a confocal laser scanning microscope (FluoView 3.1; Olympus, Japan).

### Statistical analysis.

Significant differences were determined by performing Student’s *t* test or one-way analysis of variance in GraphPad Prism 9 (GraphPad Software, CA, USA). In the figures, statistical significance or lack thereof is indicated as follows: *, *P < *0.05; **, *P < *0.01; ***, *P < *0.001; ns, not significant.
